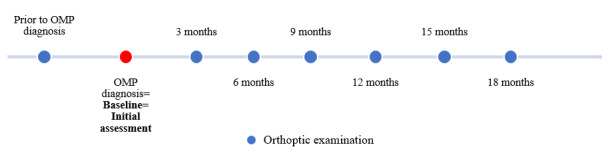# Recovery of Third, Fourth, and Sixth Cranial Nerve Palsies in Pituitary Adenoma and Meningioma Patients

**DOI:** 10.1097/WNO.0000000000001990

**Published:** 2023-09-04

**Authors:** Li-Pei J. Lu, Iris C. M. Pelsma, Friso de Vries, Saskia P. A. van Hulst-Ginjaar, Wouter R. van Furth, Marco J. T. Verstegen, Fleur L. Fisher, Amir H. Zamanipoor Najafadabadi, Nienke R. Biermasz, Stijn W. van der Meeren, Irene C. Notting

**Affiliations:** Departments of Ophthalmology (L-PJL, SPAvH-G, AHZN, SWvdM, ICN), and Neurosurgery (WRvF, MJTV, FLF, AHZN), and Division of Endocrinology and Metabolism (ICMP, FdV, WRvF, MJTV, AHZN, NRB, SWvdM, ICN), and Department of Medicine, Center for Endocrine Tumors Leiden (CETL), Leiden University Medical Center, Leiden, the Netherlands; and Department of Ophthalmology (SWvdM), Amsterdam University Medical Center, Amsterdam, the Netherlands.

## Abstract

**Background:**

This retrospective, observational cohort study aimed to determine recovery rate and recovery time of ocular motor nerve palsies (OMP) of third (CN III), fourth (CN IV), or sixth cranial nerves (CN VI)—and associated prognostic factors—in meningioma and pituitary adenoma (PA) patients.

**Methods:**

A total of 25 meningioma (28 eyes) and 33 PA patients (36 eyes), treated at the Leiden University Medical Center in the Netherlands from January 1, 1978 to January 31, 2021, were included. OMPs were evaluated according to a newly created recovery scale using on-clinical and orthoptic examinations, which were performed every 3–4 months until palsy recovery, or at 18 months follow-up.

**Results:**

Recovery rates of CN III (meningioma 23.5% vs PA 92.3%), CN IV (meningioma 20% vs PA 100%), and CN VI (meningioma 60% vs PA 100%) palsies were observed at 18 months follow-up, with differences between the 2 tumor types being observed in the treated patients only. Median recovery time of all OMPs combined was significantly longer in meningioma patients (37.9 ± 14.3 months vs 3.3 ± 0.1 months; *P* < 0.001). No significant protective or risk factors for recovery rate or time were identified.

**Conclusions:**

OMP recovery rates in treated patients were more favorable in patients with PA compared with patients with meningiomas, independent of OMP cause. With these new insights in OMP recovery, more accurate prognoses and appropriate follow-up strategies can be determined for meningioma and PA patients with OMPs.

## Abstract 2

Supplemental Digital Content is Available in the Text.

Well-coordinated ocular motility is essential for visual function and health-related quality of life.^1^ Cranial nerves responsible for ocular motility are the oculomotor nerve (CN III), trochlear nerve (CN IV), and abducens nerve (CN VI), which run through the cavernous sinus (CS) in close relation to the anterior skull base. Hence, these nerves are prone to compression by (para)sellar and anterior skull base neoplasms.^2^ Most of these tumors are pituitary adenomas (PA) and meningiomas. PA are benign tumors arising from the pituitary, accounting for 17.1% of all intracranial tumors.^3^ Meningiomas are mostly benign tumors (WHO Grade I: 80%) arising from the meninges, and represent approximately 39% of all primary intracranial tumors.^3^ Because meningiomas can originate from any location, the cranial nerves can be anatomically involved. Moreover, cranial nerve damage can occur as a complication from surgical treatment, especially when tumors invade the CS.^4–7^

Ocular motor nerve palsies (OMPs)—associated with diplopia, ptosis, mydriasis, and accommodative disorders—occur in PA and meningioma patients with a prevalence ranging from 4.5% to 17%.^4,8^ Complete or partial recovery of OMPs has been reported after (surgical) treatment, with preoperative OMPs being completely recovered or significantly improved after surgery in 70%–89% of PA patients.^9,10^ By contrast, in solely 33.3%–42.6% of meningioma patients, complete recovery was observed.^5,6^ To date, no studies have compared OMP recovery in PA and meningioma patients. Differences in the manifestation of OMP between these pathologies may provide us with insights regarding the underlying mechanisms of OMP. Accordingly, we aim to determine the difference in recovery rate and recovery time of OMP between meningioma and PA patients, and which prognostic factors predict recovery rate and recovery time in OMPs.

## METHODS

### Patients

This retrospective cohort study included meningioma and PA patients from the outpatient clinic of the Department of Ophthalmology at the Leiden University Medical Center (LUMC), a tertiary referral center for patients with these pathologies, from January 1, 1978 to January 31, 2021. The following inclusion criteria were used: patients ≥18 years with surgery- or tumor-induced OMP(s) of CN III, CN IV, and/or CN VI. Notably, multiple OMPs occurring in the same patient were counted separately. Exclusion criteria were: (I) incomplete documentation of orthoptic/ophthalmologic examination (<2 orthoptic follow-up examinations and/or incomplete follow-up because of loss to follow-up), (II) mechanical restriction of extraocular muscles because of tumor size, orbital involvement, or surgery-related edema, (III) a history of diplopia or strabismus (operations) unrelated to the tumor, and (IV) other diseases or complications causing OMPs (e.g., severe complicated diabetes mellitus [DM]). The study was approved by the LUMC Medical Ethics Committee, and a verbal declaration of nonobjection was obtained from all patients.

### Study Design

**FIG. 1.**
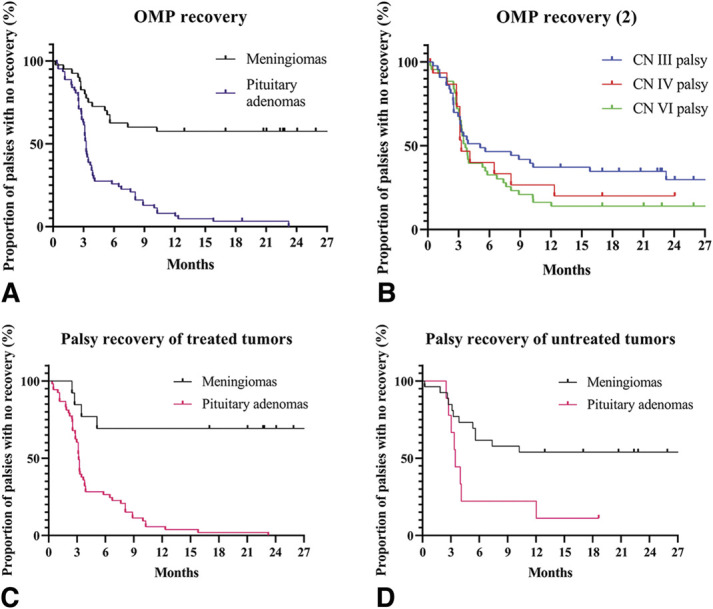
Recovery time of OMPs. OMP recovery differences between cranial nerves and between meningiomas and pituitary adenomas are shown in the Kaplan–Meier curves (data were shown as median ± SEM). **A**. OMP recovery probability between all meningioma and pituitary adenoma patients independent of affected ocular motor nerve (meningioma: 37.9 ± 14.3 vs pituitary adenoma: 3.3 ± 0.1 months, *P* < 0.001). **B**. OMP recovery for the individual cranial nerves showed a median recovery time of 5.1 ± 3.0 for CN III, 3.3 ± 0.5 for CN IV, and 3.7 ± 0.3 months for CN VI, respectively (*P =* 0.339). **C**. OMP recovery between treated meningiomas and pituitary adenomas is shown. Median recovery time in meningioma patients was 37.9 ± 24.1 months compared with 3.2 ± 0.1 months in pituitary adenoma patients (*P <* 0.001). **D**. OMP recovery between untreated meningiomas and pituitary adenomas is shown. Median recovery time of meningiomas was 37.9 ± 19.5 months and 3.5 ± 0.1 months in pituitary adenomas (*P* = 0.015). CN III, third cranial nerve; CN IV, fourth cranial nerve; CN VI, sixth cranial nerve; OMP, ocular motor nerve palsy; SEM, standard error of median.

Variables and outcomes were assessed at different time points (See **Supplemental Digital Content**, **Figure 1**, http://links.lww.com/WNO/A775). Summarizing, patients underwent follow-up until OMP recovery, or for a minimum duration of 18 months (3–4 months intervals). If no recovery was achieved within 18 months, follow-up was extended until recovery or last moment of data collection, January 31, 2021. All ophthalmic and orthoptic assessments were performed by an ophthalmologist and orthoptist during (follow-up) examinations.

### Variables

Data were retrospectively collected, and included baseline characteristics (e.g., age, gender), tumor diagnosis (including type, CS involvement, and presence of apoplexy [radiological diagnoses]), relevant medical history (e.g., DM, hypertension^11^), and treatment modalities. Notably, patients were rarely treated for the OMP specifically, because patients were treated for the cause of the OMP, that is, the meningioma or PA. Ophthalmologic evaluation included best-corrected visual acuity (BCVA) using a Snellen chart followed by logMAR conversion, and orthoptic assessment at baseline and during follow-up.

#### Orthoptic Assessment

Standard orthoptic evaluation consisted of ocular deviation and movement assessment (ODMA), duction movement assessment, and Hess screen testing. ODMAs were manually performed using the cover test in the 9 gaze directions. Duction movements were measured using a synoptophore (Clement Clarke, model 2003, Haag-Streit UK Ltd, Harlow, United Kingdom). Normal duction movements are abduction and adduction of ≥ −40 and 40°, respectively, and elevation and depression of ≥30°. In case of diplopia and adequate BCVA, Hess screen testing (Clement Clarke, Haag-Streit, UK Ltd) was manually performed and compared with the previous Hess chart. Patients were questioned for subjective diplopia. If eyes were blind/had significantly decreased vision (Snellen BCVA < 0.1), diplopia could not be assessed.

### Outcomes

A 5-tier recovery scale was created to evaluate OMP recovery, as shown in Table 1. All categories were defined using the extent of subjective diplopia, duction restrictions, restrictions on Hess charts, and misalignment of the eye(s). In case of CN III palsies, mydriasis, ptosis, and accommodative disorder were not taken into account, because eye motility has been demonstrated to be more sensitive for (prolonged) CN III recovery evaluation.^8^

**TABLE 1.** Recovery scale for ocular motor nerve palsies

Category	Criteria			
	Deviation and Movement Assessment	Duction Restrictions	Restrictions on Hess Chart	Diplopia
1: Complete recovery	None	None	None	Absent
2: Clinically relevant recovery*	Minimal*	≤5° restriction of normal ductions*	Minimal*	Minimal*
3: Partial recovery	Partial improvements from baseline restriction	Partial improvement (from baseline till 6° of normal ductions)	Partial improvements	Present/absent†
4: No change	Same as baseline restriction	Up to 2° improvement from baseline restriction	Same as baseline	Present/absent†
5: Deterioration	Worse than baseline restriction	≥3° deterioration from baseline restriction	Worse than baseline	Present/absent†

The recovery scale categories were used to evaluate OMP recovery in meningioma and pituitary adenoma patients. Normal ductions are −40°, 40°, 30° and 30° for abduction, adduction, elevation and depression, respectively. For further analyses, OMP recovery was defined as Category 1 and 2 combined.

* Objective restrictions without subjective symptoms interfering with daily activities.

† Patients with decreased vision or blind eyes did not necessarily suffer from diplopia despite substantial duction restrictions.

OMP, ocular motor nerve palsy.

OMP recovery was defined as recovery scale's Category 1 and 2 combined (Table 1). Recovery rates were determined at 6, 12, and 18 months, and 24 months of follow-up in 18 patients (11 meningioma and 7 PA patients, respectively). Recovery time was calculated from the day of OMP diagnosis until the date of the first follow-up appointment at which recovery was reported. In the absence of orthoptic examinations, clinical reports were used to evaluate OMP recovery.

### Statistics

Data—collected in Castor (Castor EDC, Amsterdam, NL)—were analyzed using IBM SPSS version 25 (SPSS Inc., Chicago, IL). Data were reported as number of patients (N), number of eyes, or palsies (n), with percentages (%) for categorical variables. Continuous variables were reported as a mean with an SD, or median with an interquartile range (IQR). Chi-square test or Fisher exact test (categorical variables), and independent-samples *t* test or Mann-Whitney *U* test (continuous variables) were used to compare patient groups (all results presented as meningioma vs PA patients). Mixed model analysis was used to determine differences in BCVA between meningiomas and PAs for OMP diagnosis and recovery. Kaplan–Meier curves were used to analyze mean OMP recovery time (expressed as median ± SEM). Differences between tumor types were calculated using a Logrank test (Mantel–Cox). To analyze predictive factors, multivariable logistic regression (recovery rate at 18 months), and Cox proportional hazards regression models (recovery time) were used. Statistical significance was set at *P* < 0.007 (false discovery rate adjusted *P*-value).^12^

## RESULTS

### Clinical Characteristics

Of 336 patients identified from pre-existing databases, 58 patients (meningiomas N = 25; PA N = 33) and 64 eyes (meningioma n = 28; PA n = 36) met all inclusion criteria (See **Supplemental Digital Content**, **Figure 2**, http://links.lww.com/WNO/A776)*.* Baseline characteristics are summarized in Table 2. Apoplexy was observed in 18/33 PA patients (54.5%). Sex (72.0% females vs 45.5% females, *P =* 0.043), age (56.6 ± 12.3 years vs 54.0 ± 16.9 years, *P =* 0.521), and cavernous sinus involvement (68.0% vs 48.5%, *P* = 0.137) did not differ significantly between the patient groups. Less patients with meningioma were treated compared with patients with PA (36.0% vs 93.9%, *P* = 0.002). Twelve PA patients were treated before OMP (surgery N = 11, RT N = 1), of which 5/12 (41.7%) resulted in surgery-related OMP, whereas nearly all meningioma patients treated with surgery (N = 10) were complicated by newly developed postoperative OMP (N = 9/10, 90%). Nevertheless, tumor compression was the primary cause of OMPs in both patient groups (67.9% vs 86.1%, *P =* 0.041).

**TABLE 2.** Baseline characteristics of meningioma and pituitary adenoma patients with ocular motor nerve palsies

Baseline Characteristics			
Patient Characteristics	Meningioma (N = 25)	Pituitary Adenoma (N = 33)	*P*
Gender (female)	18 (72.0%)	15 (45.5%)	0.043*
Age (y)	56.6 ± 12.3	54.0 ± 16.9	0.521†
Comorbidities			
Diabetes mellitus	4 (16.0%)	4 (12.1%)	0.715‡
Hypertension	4 (16.0%)	4 (12.1%)	0.715‡
Cavernous sinus involvement	17 (68.0%)	16 (48.5%)	0.137*
Apoplexy‖	NA	18 (54.5%)	NA
Location meningioma			
Petroclival	7 (28.0%)	NA	NA
Sphenoid wing	8 (32.0%)	NA	
Cavernous sinus	4 (16.0%)	NA	
Proc. Clin. anterior	2 (8.0%)	NA	
Cerebellar	2 (8.0%)	NA	
Proc. Clin. posterior	1 (4.0%)	NA	
Cerebellopontine angle	1 (4.0%)	NA	
Type of pituitary adenoma			
Nonfunctioning	NA	23 (69.7%)	
Acromegaly	NA	3 (9.1%)	
Cushing	NA	3 (9.1%)	
Prolactinoma	NA	2 (6.1%)	
Gonadotroph adenoma	NA	2 (6.1%)	

Characteristics for the Included Eyes	Meningioma (n = 28)	Pituitary Adenoma (n = 36)	*P*
BCVA at OMP diagnosis	1.0 (0.9–1.2)	0.9 (0.23–1.15)	0.033§
Cranial nerve palsy diagnosis			
Isolated CN.III	8 (28.6%)	8 (22.2%)	
Isolated CN IV	2 (7.1%)	0 (0%)	
Isolated CN VI	9 (32.1%)	9 (25.0%)	
CN III + CN VI	6 (21.4%)	8 (22.2%)	
CN III + CN IV	0 (0%)	3 (8.3%)	
CN IV + CN VI	0 (0%)	1 (2.8%)	
CN III + CN IV + CN VI	3 (10.7%)	7 (19.4%)	
Modalities of tumor treatment			
Surgery	8 (28.6%)	27 (75.0%)	<0.001*
Surgery with adjuvant RT	1 (3.6%)	0 (0.0%)	
RT	1 (3.6%)	0 (0.0%)	
Chemotherapy¶	0 (0.0%)	1 (2.8%)	
Conservative	18 (64.3%)	8 (22.2%)	
Pharmacologic	0 (0.0%)	6 (16.7%)	
OMP etiology			
Tumor-related	19 (67.9%)	31 (86.1%)	0.041*
Surgery-related	9 (32.1%)	5 (13.9%)	
Follow-up after OMP diagnosis months	13.8 (3.7–23.8)	6.3 (2.7–16.2)	0.067§

Baseline characteristics of both patient groups are shown. Because several variables differ between 2 eyes of one included patient, several characteristics are reported for of all included eyes (28 eyes in the meningioma patients vs 36 eyes in the pituitary adenoma patients).

* Differences between patient groups were analyzed with Pearson Chi-square.

† Differences between patient groups were analyzed with independent samples *t* test.

‡ Differences between patient groups were analyzed with Fisher exact Test.

§ Differences between patient groups were analyzed with Mann-Whitney *U* test.

‖ Of the 18 PA patients with apoplexy, 16 patients were treated with surgery, and 2 were treated conservatively.

¶ This patient received adjuvant pharmacologic treatment with temozolomide because of an aggressive pituitary adenoma (Cushing).

BCVA, best-corrected visual acuity; CN III, third cranial nerve; CN IV, fourth cranial nerve; CN VI, sixth cranial nerve; N, number of patients; n, number of eyes; NA, not assessed; OMP, ocular motor nerve palsy; RT, radiotherapy.

### Diagnosis and Distribution of Ocular Motor Nerve Palsies

In the described 64 eyes, 102 OMPs were observed, of which the distribution is shown in Table 2. An isolated CN VI palsy was the most frequently occurring OMP in both tumor groups (32.1% vs 25.0%). Within the 28 eyes of meningioma patients, 40 palsies were observed, involving CN III in 17 (42.5%), CN IV in 5 (12.5%), and CN VI in 18 (45%) eyes. PA patients' eyes had 62 palsies in total, with 26 CN III (41.9%), 11 CN IV (17.7%), and 25 CN VI palsies (40.3%).

### Recovery of Ocular Motor Nerve Palsies

Recovery was observed in 78/102 palsies (76.5%). In meningioma patients, complete recovery (Category 1) was observed in 3/40 (7.5%) palsies, compared with 34/62 (54.8%) palsies in PA patients. Furthermore, clinical recovery (Category 2) was observed in 15/40 (37.5%) OMPs in meningioma patients, compared with 26/62 (42%) OMPs in PA patients.

#### Recovery Rates of Ocular Motor Nerve Palsies

Recovery rates for the different follow-up periods are shown in Table 3*.* At 18 months, recovery rates for all OMPs were significantly lower in treated meningioma patients compared with treated PA patients: CN III 37.5% vs 95.8%, *P* = 0.0015; CN IV 0% vs 100%; and CN VI 40% vs 100%, *P* = 0.007, respectively. No differences between the 2 untreated tumor groups were observed.

**TABLE 3.** Recovery rates of ocular motor nerve palsies in meningioma and pituitary adenoma patients

		Total			Treated*	Untreated			
		Meningioma (17 Palsies)	Pituitary Adenoma (26 Palsies)	Meningioma (8 Palsies)	Pituitary Adenoma (24 Palsies)	*P* †	Meningioma (9 Palsies)	Pituitary Adenoma (2 Palsies)	*P* ‡
CN III	6 mo	4 (23.5%)	19 (73.1%)	3 (37.5%)	18 (75%)		1 (11.1%)	1 (50%)	
	12 mo	4 (23.5%)	23 (88.5%)	3 (37.5%)	22 (91.7%)		1 (11.1%)	1 (50%)	
	18 mo	4 (23.5%)	24 (92.3%)	3 (37.5%)	23 (95.8%)	0.0015§	1 (11.1%)	1 (50%)	0.325§
	24 mo	5 (29.4%)	25 (96.2%)	4 (50%)	24 (100%)		1 (11.1%)	1 (50%)	

		Meningioma (5 Palsies)	Pituitary Adenoma (11 Palsies)	Meningioma (1 Palsies)	Pituitary Adenoma (10 Palsies)		Meningioma (4 Palsies)	Pituitary Adenoma (1 Palsies)	
CN IV	6 mo	1 (20%)	8 (72.7%)	0 (0%)	7 (70%)		1 (25%)	1 (100%)	
	12 mo	1 (20%)	10 (90.9%)	0 (0%)	9 (90%)		1 (25%)	1 (100%)	
	18 mo	1 (20%)	11 (100%)	0 (0%)	10 (100%)	NA	1 (25%)	1 (100%)	0.534§
	24 mo	1 (20%)	11 (100%)	0 (0%)	10 (100%)		1 (25%)	1 (100%)	

		Meningioma (18 Palsies)	Pituitary Adenoma (25 Palsies)	Meningioma (5 Palsies)	Pituitary Adenoma (19 Palsies)		Meningioma (13 Palsies)	Pituitary Adenoma (6 Palsies)	
CN VI	6 mo	10 (55.6%)	19 (76%)	2 (40%)	14 (73.7%)		8 (61.5%)	5 (83.3%)	
	12 mo	12 (66.7%)	24 (96%)	2 (40%)	19 (100%)		10 (76.9%)	5 (83.3%)	
	18 mo	12 (66.7%)	25 (100%)	2 (40%)	19 (100%)	0.007§	10 (76.9%)	6 (100%)	0.353§
	24 mo	13 (72.2%)	25 (100%)	2 (40%)	19 (100%)		11 (84.6%)	6 (100%)	

Recovery rates of OMPs at 6, 12, 18, and 24 months in meningioma and pituitary adenoma patients are shown.

* Treated indicates treated for the tumor with palsy.

† *P*-value of recovery rates between treated meningiomas and pituitary adenomas with palsy.

‡ *P*-value of recovery rates between untreated meningiomas and pituitary adenomas with palsy.

§ Differences between tumor groups and treatment groups were analyzed with Fisher exact test for the main outcome point (i.e., 18 months).

CN III, third cranial nerve; CN IV, fourth cranial nerve; CN VI, sixth cranial nerve; NA, not applicable; OMP, ocular motor nerve palsy.

Recovery rates (both tumor types combined) were observed to be highest for CN VI palsies (84.1%) and lowest for CN III palsies (65.1%). Recovery after 18 months was still observed in 2 meningioma patients' eyes (one with a CN III palsy, and one with a CN VI palsy), compared with one eye in a PA patient with a CN III palsy. Recovery rates of tumor-induced OMP did not differ significantly from surgery-related OMP (*P =* 0.460). Moreover, in PA patients, no differences in recovery rates were observed between the nonapoplexy and apoplexy group (*P =* 1.00).

#### Best-Corrected Visual Acuity Recovery and Ocular Motor Nerve Palsy Recovery

Median BCVA at baseline did not significantly differ between meningioma and PA patients (1.0 (0.9–1.2) vs 0.9 (0.23–1.15), *P* = 0.039; logMAR 0.00 (−0.08 to 0.05) vs 0.03 (−0.08 to 0.52), *P* = 0.067). When OMP recovered or follow-up was discontinued, BCVA improvement was greater in PA patients compared with meningioma patients (tumor type: *P* = 0.011, time: *P* < 0.001, tumor × time: *P* < 0.001) (See **Supplemental Digital Content**, **Figure 3**, http://links.lww.com/WNO/A777).

#### Prognostic Factors for Recovery Rate

Following correction for age, sex, hypertension, and DM, no prognostic factors were found for CN III and CN VI palsies, as reported in Table 4. Within meningioma patients with CN IV palsy, only one patient recovered, and therefore no comparative and regression analyses were performed.

**TABLE 4.** Prognostic factors for recovery rate and recovery time in the study population at 18 months

	Recovery Rate						Recovery Time					
	CN III			CN VI			CN III			CN VI		
	OR	95% CI	*P*	OR	95% CI	*P*	HR	95% CI	*P*	HR	95% CI	*P*
Age (y)	1.004	0.908–1.110	0.934	1.035	0.936–1.144	0.504	0.990	0.964–1.017	0.455	1.001	0.979–1.023	0.959
Sex*	0.639	0.022–18.470	0.794	4.647	0.265–81.467	0.293	0.985	0.423–2.296	0.937	2.053	0.730–5.773	0.173
Tumor type†	0.019	0.001–0.369	0.009	0.000	0.000–ꝏ	0.998	1.871	0.543–6.445	0.321	0.938	0.324–2.714	0.906
Treatment‡	0.013	0.000–1.574	0.076	30.236	0.624–1,464.018	0.085	0.258	0.024–2.762	0.263	1.336	0.443–4.031	0.607
Hypertension	0.112	0.002–7.349	0.305	6.662	0.289–153.821	0.236	0.52	0.134–2.017	0.344	1.028	0.407–2.598	0.954
Diabetes mellitus	0.143	0.003–6.810	0.324	0.163	0.003–8.099	0.362	0.423	0.096–1.874	0.257	0.393	0.121–1.269	0.118

Prognostic factors for recovery rate and recovery time were assessed in CNII and CN VI ocular motor nerve palsy in the total study population. Logistic multivariate regression model and Cox regression model was used to analyze the significance of variables.

* Reference sex was male sex.

† Tumor type was defined as meningioma vs pituitary adenoma patients.

‡ Treatment was defined as treated vs untreated patients.

CI, confidence interval; CN III, third cranial nerve; CN VI, sixth cranial nerve; HR, hazard ratio; OR, odds ratio.

#### Recovery Time of Ocular Motor Nerve Palsies

Kaplan–Meier curves showing OMP recovery of meningioma and PA patients are presented in Figure 1. Median recovery time of all OMPs combined was significantly longer in meningioma patients compared with PA patients (37.9 ± 14.3 vs 3.3 ± 0.1 months, *P* < 0.001, Fig. 1A). No significant difference in median recovery time was observed between the 3 cranial nerves, as shown in Figure 1B (CN III: 5.1 ± 3.0, CN IV: 3.3 ± 0.5, CN VI: 3.8 ± 0.3 months, *P =* 0.339). Furthermore, palsies of treated PAs recovered faster than palsies of treated meningiomas (*P* < 0.001, Fig. 1C), whereas no significant difference was observed in the untreated group (*P* = 0.015, Fig. 1D). Median recovery time of tumor-induced OMPs did not differ substantially from surgery-related OMPs (3.8 ± 0.7 vs 4.0 ± 2.5 months, *P =* 0.823), and no differences in recovery time was observed between the nonapoplexy and apoplexy group of PA patients (*P =* 0.798). As shown in Table 4, no prognostic factors were found for recovery time of CN III and CN VI palsies.

## DISCUSSION

Our study demonstrates that OMP recovery was more favorable in PA compared with meningioma patients. Virtually, all OMP in patients with PA recovered partially or completely after 18 months, compared with less than half of OMP in patients with meningioma. The difference between these 2 patient groups was achieved in the treated patients. Clinical recovery was occasionally identified after 18 months, reflecting the potential of OMP recovery after prolonged follow-up.

The observed higher OMP recovery rates and concomitant BCVA recovery in patients with PA compared with patients with meningioma are in line with previous studies,^5,6,8–10,13,14^ which could hypothetically be explained by apoplexy, or differences in treatment strategies. As expected, apoplexy was observed solely in patients with PA, because these tumors have high metabolic demands and poor vascular densities.^15^ Meningiomas are often slow-growing and highly vascularized tumors, often requiring more invasive surgery compared with PAs.^16^ Moreover, OMP in apoplectic patients with PA has been reported to be more likely to recover than OMP in nonapoplectic patients.^13^ Apoplexy could, therefore, explain the higher recovery rates in patients with PA. Surprisingly, recovery rates were not affected by the presence of apoplexy in this study.

A significant disparity in treatment modalities for the tumor types was observed. Total resection rates have been reported to be similar in patients with meningioma and PA.^17,18^ However, 80%–90% of the meningiomas in those studies were not complicated by CS invasion, whereas most meningioma patients had CS invasion in this study (i.e., 68%).^6,16^ Treated meningioma patients had lower recovery rates than treated patients with PA in this study. Unexpectedly, treatment did not influence recovery rates after correction for potential confounding factors. However, regarding recovery rate and recovery time, a distinction should be made between the tumor-induced OMPs and surgery-related OMPs, because treatment would only benefit tumor-induced OMPs. Early treatment in tumor-induced OMPs has been identified as favorable for recovery rate in CN III palsies.^13^ Early treatment in meningiomas is often not achievable because of the insidious disease onset, resulting in treatment delay.^16^ Consequently, treatment timing in meningiomas remains to be systematically investigated.

Next to greater recovery rates, substantially shorter recovery times were observed in patients with PA compared with meningioma. Most OMP recoveries occurred within 6 months, with the prospect of recovery thereafter being rare in patients with meningioma, as published previously.^6,8,14^ Several differences between patients with PA and meningioma should be considered regarding the disparity in recovery time: tumor growth rate and patterns, affected cranial nerves, and OMP etiology. First, meningiomas tend to cause prolonged cranial nerve compression compared with PAs, resulting in delayed recovery, or no recovery at all, despite tumor treatment.^6,7^ Second, because CN IV has an anatomical predisposition to be affected by tumor compression (long, thin nerve), and CN VI is most likely to be damaged during surgery (because of its location close to skull base), we would expect that these nerves would have the longest recovery times.^6,19^ However, no statistically significant differences in recovery times were observed between the different cranial nerves, because the number of patients in this study was not large enough to reach statistical significance. Moreover, apoplexy-induced OMPs (solely occurring in PA patients) would recover faster with adequate surgical intervention compared with other OMPs because of the acute onset,^8^ a finding not observed in the present study.

Several limitations of the present study must be considered. First, the sample size of our study was limited, because OMPs are uncommon manifestations in patients with meningioma and PA.^4,8^ Second, previous studies adopted different criteria and definitions for recovery, hampering comparison between studies.^6,20^ In future studies, clinical recovery, in addition to complete and partial recovery, should be included, that is, using the proposed recovery scale (Table 1). Despite patients with meningioma being less likely to recover completely, a significant percentage of these patients reached the clinical recovery state, implying that OMP diagnosis no longer resulted in impairments in daily life in these patients. In addition, in-depth analyses (e.g., recovery time, prognostic factors) of CN IV palsies in patients with meningioma were not performed, because only one patient's OMP recovered.

In conclusion, this retrospective study showed that OMP recovery rates were more favorable in PA patients compared with meningioma patients independent of OMP etiology, especially in treated patients. Moreover, OMPs in PA patients recovered faster than OMPs in patients with meningioma. With these new insights in OMP recovery rates and times, physicians can provide more accurate prognoses, and therefore more appropriate follow-up strategies for patients with OMP caused by meningioma or PA.

STATEMENT OF AUTHORSHIP

Conception and design: L.-P. J. Lu, I. C. M. Pelsma, F. de Vries, S. W. van der Meeren, I. C. Notting; Acquisition of data: L.-P. J. Lu, I. C. M. Pelsma, F. de Vries, A. H. Zamanipoor Najafadabadi, S. W. van der Meeren, I. C. Notting; Analysis and interpretation of data: All authors. Drafting the manuscript: L.-P. J. Lu, I. C. M. Pelsma, F. de Vries, S. W. van der Meeren, I. C. Notting; Revising the manuscript for intellectual content: All authors. Final approval of the completed manuscript: All authors.

## Supplementary Material

**Figure:**
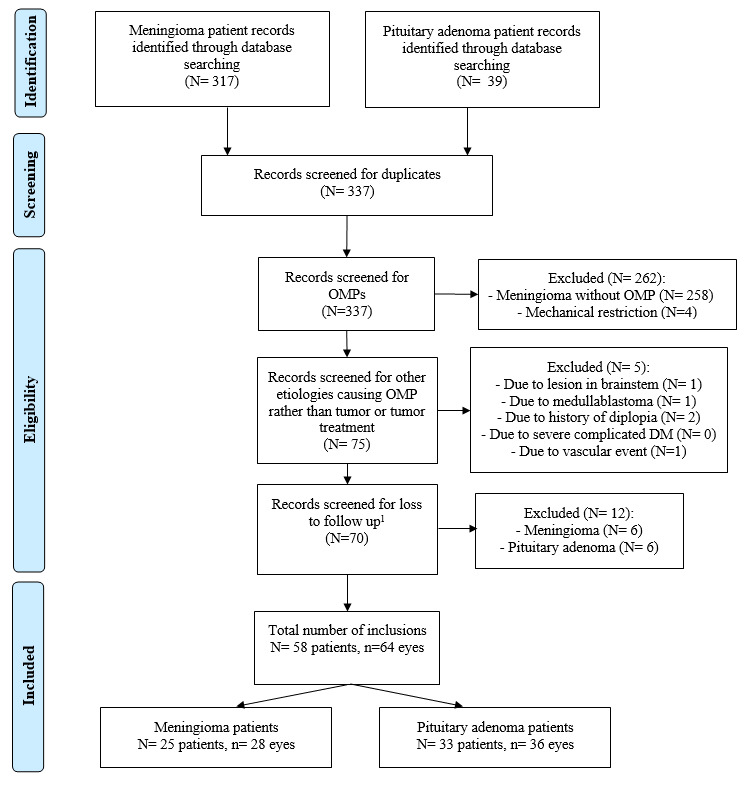


**Figure:**
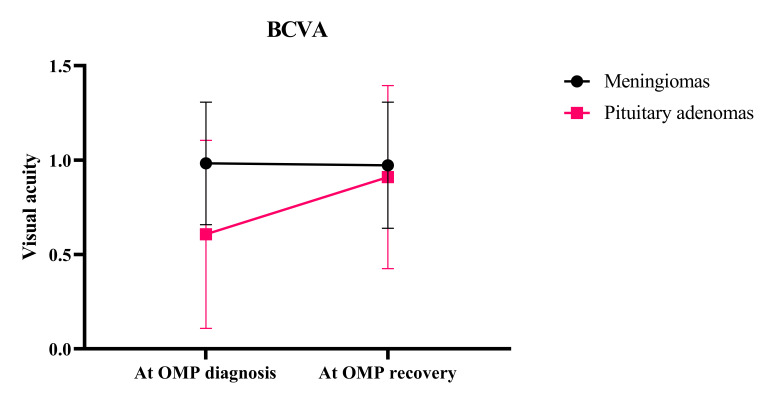


**Figure:**